# Clinical Gestalt to Predict Bacterial Infection and Mortality in Emergency Department Patients: A Prospective Observational Study

**DOI:** 10.1007/s11606-025-09440-7

**Published:** 2025-02-26

**Authors:** Tanguy Espejo, Ricardo Nieves-Ortega, Livia Amsler, Henk Börje Riedel, Gianmarco Balestra, Christiane Rosin, Christoph Becker, Kriemhild Lippay, Christian Hans Nickel, Roland Bingisser

**Affiliations:** 1 Emergency Department, University Hospital Basel, University of Basel, Basel, Switzerland; 2 Department of Cardiology, University Hospital Basel, University of Basel, Basel, Switzerland; 3 Department of Psychosomatic Medicine, University Hospital Basel, University of Basel, Basel, Switzerland

**Keywords:** Clinical gestalt, Infection, Emergency department, Antibiotics, Sepsis

## Abstract

**Background:**

Time to treatment is a significant predictor of mortality in emergency department (ED) patients with bacterial sepsis. Strategies for the early detection of bacterial infection and sepsis are lacking. Clinical gestalt is a tool for assessing and synthesizing the entire clinical picture, focusing on the first clinical impression at presentation.

**Objective:**

This study aimed to assess ED physicians’ clinical gestalt for the prediction of bacterial infection and mortality in ED patients presenting with signs and symptoms of infection.

**Design:**

Prospective, observational study with a 30-day follow-up.

**Participants:**

Patients aged 18 or older presenting to the ED with signs and symptoms compatible with an infection and abnormal vital signs were included.

**Main Measures:**

ED physicians recorded their clinical gestalt using a visual analog scale (VAS) to assess the likelihood of bacterial infection and responded to a dichotomous question regarding the probability of a patient’s death. The main outcome was the confirmed diagnosis of an acute bacterial infection. Final diagnoses, based on laboratory and follow-up information, were adjudicated by an expert panel.

**Key Results:**

In total, 444 patients were included. Median age was 68 years [IQR 51, 80] and median National Early Warning Score (NEWS) was 5 [IQR 3, 7]. Median VAS for physicians’ clinical gestalt regarding bacterial infection likelihood was 8.2 [IQR 6.7, 9.0] of 10 in patients with bacterial infection, 2.3 [IQR 1.2, 4.3] in patients with viral infection, 4.6 [IQR 4.0, 7.3] in patients with an infection due to another pathogen, and 2.3 [IQR 1.1 6.2] in patients with no acute infection (*p*-value = <0.001). Clinical gestalt’s sensitivity regarding 30-day mortality was 57.1% [95%CI 37.2 to 75.5] with a specificity of 83.4% [95%CI 79.7 to 86.9].

**Conclusion:**

In this study of ED patients presenting with signs and symptoms of infection, clinical gestalt was shown to be useful differentiating between bacterial and infections of other causes. Antibiotic prescription rate increased with the likelihood of bacterial infection according to physician gestalt. Lastly, simple heuristic prognostication of mortality (likely vs. unlikely) carried some, but limited, prognostic value.

**Supplementary Information:**

The online version contains supplementary material available at 10.1007/s11606-025-09440-7.

## INTRODUCTION

Serious infections account for 2 to 3% of all ED presentations.^1,2^ A fast and accurate diagnosis is crucial to prevent complications and reduce sepsis-related mortality.^3,4^ Importantly, an early differentiation between bacterial, viral, and other causes of infection is the prerequisite for antimicrobial treatment and may help to prevent overuse of antibiotics.^5^ Unfortunately, symptoms, clinical signs, and inflammatory markers do not permit early and reliable identification of bacterial infections.^6–8^ Inflammatory markers, such as C-reactive protein (CRP) and procalcitonin (PCT), are often used to rule in or rule out a suspected bacterial infection. However, inflammatory markers generally have only moderate sensitivity and specificity regarding bacterial infection and sepsis.^9–15^ Current guidelines therefore recommend the use of tools, such as the National Early Warning Score (NEWS), the Modified Early Warning Score (MEWS), or systemic inflammatory response syndrome (SIRS) criteria as screening tools for sepsis and septic shock despite their lack of specificity for the diagnosis of bacterial infection or sepsis.^16^

Some of these scores require additional information, such as blood tests, which are not readily available at presentation. Clinical gestalt, on the other hand, is a way of assessing and gauging the entire information, focusing on the subjective clinical impression. This first clinical impression is immediately available at presentation but may differ considerably between individual clinicians.^17–19^ Gestalt typically relies on heuristics, based on experience and clinical perception in the absence of diagnostic testing (clinical chemistry and imaging).^20,21^ In the ED, physicians’ clinical gestalt was shown to be useful at triage and for outcome prediction.^22–25^ In patients with sepsis, clinical gestalt was associated with the severity of sepsis and the disposition (ICU vs. general ward admission).^26,27^

As time to treatment is a predictor of increased mortality in ED patients with sepsis tools supporting early decisions regarding bacterial infection and sepsis are urgently needed.^28,29^ Clinical gestalt has been used for many years, but, to our knowledge, there are no data on the formal use of gestalt regarding bacterial infection or mortality prediction in patients presenting to the ED with symptoms suggestive of infection.

Therefore, the aim of this study was to assess the value of ED physicians’ clinical gestalt for prediction of bacterial infection and mortality in patients presenting with signs and symptoms of infection.

## METHODS

### Study Design

This study is based on the prospective and observational “Basel Early Diagnosis of Sepsis” (BEDS) cohort with a 30-day follow-up. It was carried out in the ED of the University Hospital Basel in Northwestern Switzerland. The University Hospital Basel is an urban 700-bed primary and tertiary care center with a yearly ED patient volume of approximately 55,000 patients aged 16 years and older. Obstetric, pediatric, and ophthalmologic patients are treated in separate facilities nearby. Patient recruitment was performed from May 5, 2017, to May 31, 2018. The study was approved by the local Ethics Committee (EKNZ No. 2017-00092) and conducted according to the principles of the Declaration of Helsinki. Written informed consent was necessary for inclusion. If patients were incapable of giving informed consent, a surrogate consent could be provided by a proxy or by a physician unaffiliated to the study if no proxy was available.

### Selection of Participants

All patients aged 18 or older presenting to the ED of the University Hospital Basel between 8 am and 8 pm on weekdays were screened for inclusion by a study team of trained study physicians. Patients were eligible for inclusion if they presented with signs and symptoms compatible with an infection (identified using the screening algorithm included in the 2016 Emergency Department Sepsis Screening Tool for non-pregnant adults 12 years and over^4^ proposed by the UK Sepsis Trust in accordance with the 2016 NICE sepsis guideline^30^) and abnormal vital signs, defined as a NEWS of ≥ 2.^31^

### Data Collection

Patients were routinely triaged using the German version of the Emergency Severity Index (ESI).^32,33^ The study team then approached eligible patients and asked for informed consent. Patients’ baseline characteristics were collected by the study team at presentation. Demographics, vital signs, and the presumptive diagnosis were recorded in the electronic health record (EHR). The attending physician in charge recorded clinical gestalt regarding the likelihood of bacterial infection using a visual analog scale (VAS; 0–10) based on the following question: “What is the probability of bacterial infection in this patient?” Regarding the probability of mortality, the following question was asked: “Do you think the patient has a >50% chance of dying in the next 30 days?” This information was collected after the first contact with the patient, before any information on clinical chemistry and imaging were available. The order of operations in our department is registration, triage (including assessment of vital signs in all patients, according to ESI 5^th^ version), work-up according to the triage-nurses order-sets, short assessment by the attending physician in charge (when the VAS for clinical gestalt had to be taken down), followed by formal and structured assessment by the residents. At the timepoint of study inclusion, attending physicians did not have access to clinical chemistry or imaging data, but were aware of the patients’ history taken down by triage nurses. The extent of the initial evaluation varied, with most attending physicians conducting only a focused assessment. Our study aimed at an independent assessment of clinical gestalt. Attending physicians were therefore asked to put down their clinical judgement on the VAS away from the patients’ beds. Because it is virtually impossible to conceal vital parameters, we only included patients with abnormal vital parameters and NEWS was concealed for the study. Data were recorded in electronic case report forms (eCRF) (secuTrial®, InterActive Systems GmbH, Berlin, Germany). To gather data on diagnostic tests, antibiotic treatments, and adverse outcomes, the study database was matched with the hospital’s EHR. Follow-up regarding disposition and mortality was performed after day 30 after ED presentation using the EHR (re-presentations to the hospital), the official civil registry, and telephone interviews with primary care providers, patients, and proxies.

### Blood Samples

A venous blood sample was obtained from each participant at the time of inclusion to measure levels of inflammatory markers. Differential peripheral blood counts were performed using flow cytometry. Serum CRP was measured using a turbidimetric assay (Dade Behring, Paris, France) with a detection limit of 0.3 mg/L. Serum PCT was measured using an electrochemiluminescence immunoassay (Brahms Diagnostica GmbH, Berlin, Germany) with a detection limit of 0.02 ng/mL.

### Outcomes

#### Infection Status Adjudication

The primary outcome was the confirmed diagnosis of an acute infection, classified as bacterial, viral, or other (e.g., fungal or parasitic infection). An infection was considered acute if first symptoms occurred less than a week before presentation and if it was diagnosed within 48 h of ED presentation. For every patient, the infection category was adjudicated in a multi-step process: first, all microbiological tests obtained within 48 h of presentation were collected. These tests included microbiological cultures, polymerase chain reaction (PCR) assays, and serologic and antigen detection tests. Cases with positive microbiological tests were assessed by the study team. If the tests results aligned with the patient’s clinical diagnosis (e.g., pneumonia with positive blood culture for *Streptococcus pneumoniae*), the case was categorized accordingly with “high certainty.” Remaining cases (e.g., no positive microbiological test or typical signs of contamination) were then assessed using a modified Delphi process described below. A chart abstraction using a standardized abstraction protocol was carried out by a trained physician, blinded to the study’s hypothesis. Abstracted charts consisted of triage forms, vital signs, laboratory tests, patient reports, follow-up letters, and autopsy reports and were given to the adjudicators. An expert panel consisting of three physicians, board-certified in intensive care, infectious disease, and emergency medicine, independently reviewed charts, and classified participants into four groups: “acute bacterial infection,” “acute viral infection,” “acute infection of other (non-bacterial and non-viral) causes,” and “no acute infection.” Patients with simultaneous bacterial and viral infections were classified as “acute bacterial infection.” Adjudicators were blinded to the study’s inflammatory markers levels, to the physicians’ clinical gestalt, and to the research question. If all experts agreed, the case was categorized accordingly (concordant case). If one of three experts disagreed (discordant case), the case was adjudicated by a fourth expert. Concordant cases were also considered as “high certainty.”

#### Antibiotic Treatment

Antibiotic treatment was defined as at least one dose of an antibiotic in the first 24 h after presentation.

#### 30-Day Mortality

The time to death was defined as days between study inclusion and death.

### Statistical Analysis

Data are presented as frequencies and percentages for categorical variables and as mean with standard deviation (SD) or median with interquartile range (IQR), as appropriate, for continuous variables. Differences were tested using the Kruskal–Wallis test or the exact Fisher test, where appropriate. We assessed interrater reliability (IRR) for infection adjudication by the expert panel using the Fleiss kappa coefficient. Kappa coefficients were considered as follows: <0.20 was considered as poor agreement, 0.20 to 0.40 as fair agreement, 0.41 to 0.60 as moderate agreement, 0.61 to 0.80 as good agreement, and >0.80 as very good agreement.^34^ We assessed the performance of physicians’ clinical gestalt, inflammatory markers, NEWS, and ESI in respect to bacterial infection using receiver operator characteristic (ROC) curves, adjusted for age and sex. After adjustment for age and sex, the performance of clinical gestalt, NEWS, and ESI to predict 30-day mortality was assessed using ROC curves. Areas under the curve (AUCs) with 95% confidence interval (CI) were computed. One-to-one AUC comparisons using DeLong’s test with a 5% alpha-level (two sided) were carried out. A sensitivity analysis including only “high-certainty” cases was performed. For mortality prediction, we calculated sensitivity, specificity, positive and negative likelihood ratios (LR), and positive and negative predictive values (PV). The odds ratios (OR), unadjusted and adjusted for age and sex, from logistic regression models, were calculated for prediction of bacterial infection, antibiotic treatment, and 30-day mortality. The statistical analyses were performed using R, version 4.2.2.^35^

## RESULTS

### Population

**Figure 1:**
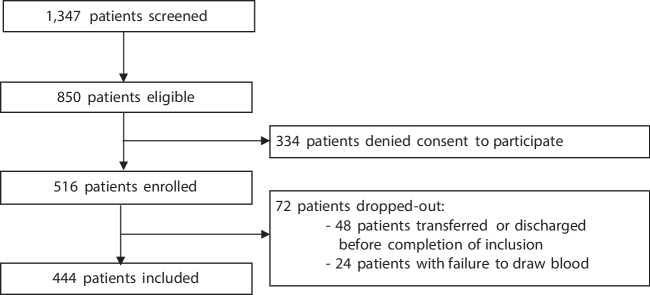
Flowchart of the study population. The chart displays recruitment procedure of ED patients presenting with history and signs compatible with an acute infection, and a National Early Warning Score (NEWS) of ≥ 2.

Of the 1347 screened patients, 850 were eligible for inclusion. Of those, 334 patients denied consent to participate and 72 dropped out due to early discharge or transfer, or due to failure to obtain a study blood sample. This resulted in a study population of 444 patients (Fig. 1). Median age was 68 years [IQR 51, 80], 195 (44%) were female, and the median NEWS was 5 [IQR 3, 7]. The most frequently assigned triage score was ESI level 2 (*n* = 248, 56%). Most patients had suspected respiratory infection (*n* = 241, 54.3%), followed by suspected urinary tract infection (*n* = 71, 16.0%). Three hundred eighty-four patients (86.5%) were admitted to the hospital, of which 39 (8.8%) to the ICU. Three patients (0.7%) were lost to follow-up at day 30. Within 30 days, 29 patients (6.6%) died (Table 1).

**Table 1:** Baseline Characteristics

Baseline characteristics (*n* = 444)		
Age, median [IQR]		68 [51, 80]
Female sex, *n* (%)		195 (44%)
ESI triage level		
	ESI 1, *n* (%)	6 (1.4%)
	ESI 2, *n* (%)	248 (56.0%)
	ESI 3, *n* (%)	184 (41.5%)
	ESI 4,* n* (%)	5 (1.1%)
	ESI 5, *n* (%)	0 (0.0%)
NEWS, median [IQR]		5 [3, 11]
Suspected infection at admission		
	Respiratory, *n* (%)	241 (54.3)
	Urinary, *n* (%)	71 (16.0)
	Abdominal, *n* (%)	62 (14.0)
	Skin, *n* (%)	26 (5.9)
	CNS, *n* (%)	14 (3.2)
	Bloodstream, *n* (%)	4 (0.9)
	Other, *n* (%)	14 (3.2)
	Unclear, *n* (%)	64 (14.4)
Inflammatory markers		
	Serum CRP (mg/l), median [IQR]	56.8 [20.8, 151.9]
	Serum Procalcitonin (ng/ml), median [IQR]	0.2 [0.1, 0.5]
	Leucocyte count (×10^9^/l), median [IQR]	10.6 [7.7, 14.0]
Physicians’ clinical gestalt regarding bacterial infection likelihood (VAS, 0–10), median [IQR]		6.7 [2.4, 8.5]
Physicians’ clinical gestalt regarding probability of patient’s death in the next month, *n* (%)		82 (19.1)
Antibiotic treatment, *n* (%)		275 (61.9)
Disposition		
	Admission to hospital (normal ward),* n* (%)	345 (77.7)
	ICU admission, *n* (%)	39 (8.8)
	Discharged to home, *n* (%)	60 (13.5)
30-day mortality, *n* (%)		29 (6.6)
Patients lost to follow-up, *n* (%)		3 (0.7)

Baseline characteristics for the study population. Data are reported as median [IQR] or *n* (%)

*Abbreviations*: *CRP*, C-reactive protein; *ESI*, Emergency Severity Index; *ICU*, intensive care unit; *IQR*, interquartile range; *NEWS*, National Early Warning Score; *VAS*, visual analog scale

### Infection Category

The infection category was adjudicated for positive microbiological tests corroborating clinical diagnosis in 201 cases (45.3%); in 174 cases (39.2%), infection status was adjudicated by expert panel consensus (concordant cases). The remaining 69 discordant cases (15.5%) were adjudicated by a fourth expert (Appendix Table 4). Adjudication process by the expert panel showed a good IRR (kappa coefficient = 0.66; 95%CI 0.61 to 0.71).

In 76 patients (17.1%), no acute infection was found. Out of the 368 patients with acute infection, the causes were bacterial in 252 (68.5%) and viral in 113 (30.7%), and 3 (0.8%) were caused by other organisms. Most of the infections were respiratory (*n* = 205, 55.7%), followed by urinary tract infection (*n* = 76, 20.6%) and abdominal infection (*n* = 43, 11.7%) (Table 2).

**Table 2:** Comparison of Patients by Infection Status

	No acute infection	Acute infection			*P*-value
		Bacterial	Viral	Other	
*n* (%)	76 (17.1)	252 (56.8)	113 (25.5)	3 (0.7)	
Age, median [IQR]	69 [51, 79]	72 [56, 83]	60 [44, 75]	75 [69, 80]	<0.001
Female sex, *n* (%)	31 (41)	106 (42)	58 (51)	0 (0)	0.138
NEWS, median [IQR]	4 [2, 6]	5 [3, 8]	5 [3, 6]	2 [2, 3]	<0.001
ESI triage level, median [IQR]	2 [2, 3]	2 [2, 3]	2 [2, 3]	2 [2, 3]	0.489
Confirmed infection					
Respiratory infection, *n* (%)		114 (45.2)	89 (78.8)	2 (66.7)	<0.001
Urinary tract infection, *n* (%)		76 (30.2)	0	0	<0.001
Abdominal infection, *n* (%)		32 (12.7)	11 (9.7)	0	0.587
Bloodstream infection, *n* (%)		34 (13.5)	1 (0.9)	0	0.001
Skin infection, *n* (%)		20 (8.0)	0	0	0.008
CNS infection,* n* (%)		0	4 (3.5)	0	0.01
Other infection, *n* (%)		10 (4.0)	1 (0.9)	0	0.266
Unclear infection focus, *n* (%)		16 (6.3)	10 (8.8)	1 (33.3)	0.481
Outcomes					
Hospitalization, *n* (%)	63 (82.9)	231 (91.7)	87 (77.0)	3 (100.0)	0.001
Length of hospital stay in days, median (IQR)	9 [5, 14]	8 [5, 15]	6 [3, 12]	9 [8, 19]	0.147
Admission to ICU, *n* (%)	10 (13.2)	21 (8.3)	8 (7.1)	0 (0.0)	0.462
7-day mortality, *n* (%)	2 (2.6)	11 (4.4)	1 (0.9)	0 (0.0)	0.074
30-day mortality, *n* (%)	4 (5.3)	23 (9.1)	2 (1.8)	0 (0.0)	0.056
Physicians’ clinical gestalt regarding bacterial infection likelihood, VAS (0–10)	2.3 [1.1, 6.2]	8.2 [6.7, 9.0]	2.3 [1.2, 4.3]	4.6 [4.0, 7.3]	<0.001
Antibiotic treatment, *n* (%)	16 (21.1)	226 (89.7)	31 (27.4)	2 (66.7)	<0.001

Data are reported as median [IQR] or *n* (%)

*Abbreviations*: *CNS*, central nervous system; *ESI*, Emergency Severity Index; *ICU*, intensive care unit; *IQR*, interquartile range; *NEWS*, National Early Warning Score; *VAS*, visual analog scale

### Prediction of Bacterial Infection

The median VAS for ED physicians’ clinical gestalt regarding bacterial infection likelihood was 8.2 [IQR 6.7, 9.0] of 10 in the bacterial infection group, 2.3 [IQR 1.2, 4.3] of 10 in the viral infection group, 4.6 [IQR 4.0, 7.3] of 10 in the other pathogens group, and 2.3 [IQR 1.1, 6.2] of 10 in the group with no acute infection (*p*-value = <0.001) (Table 2).

**Figure 2:**
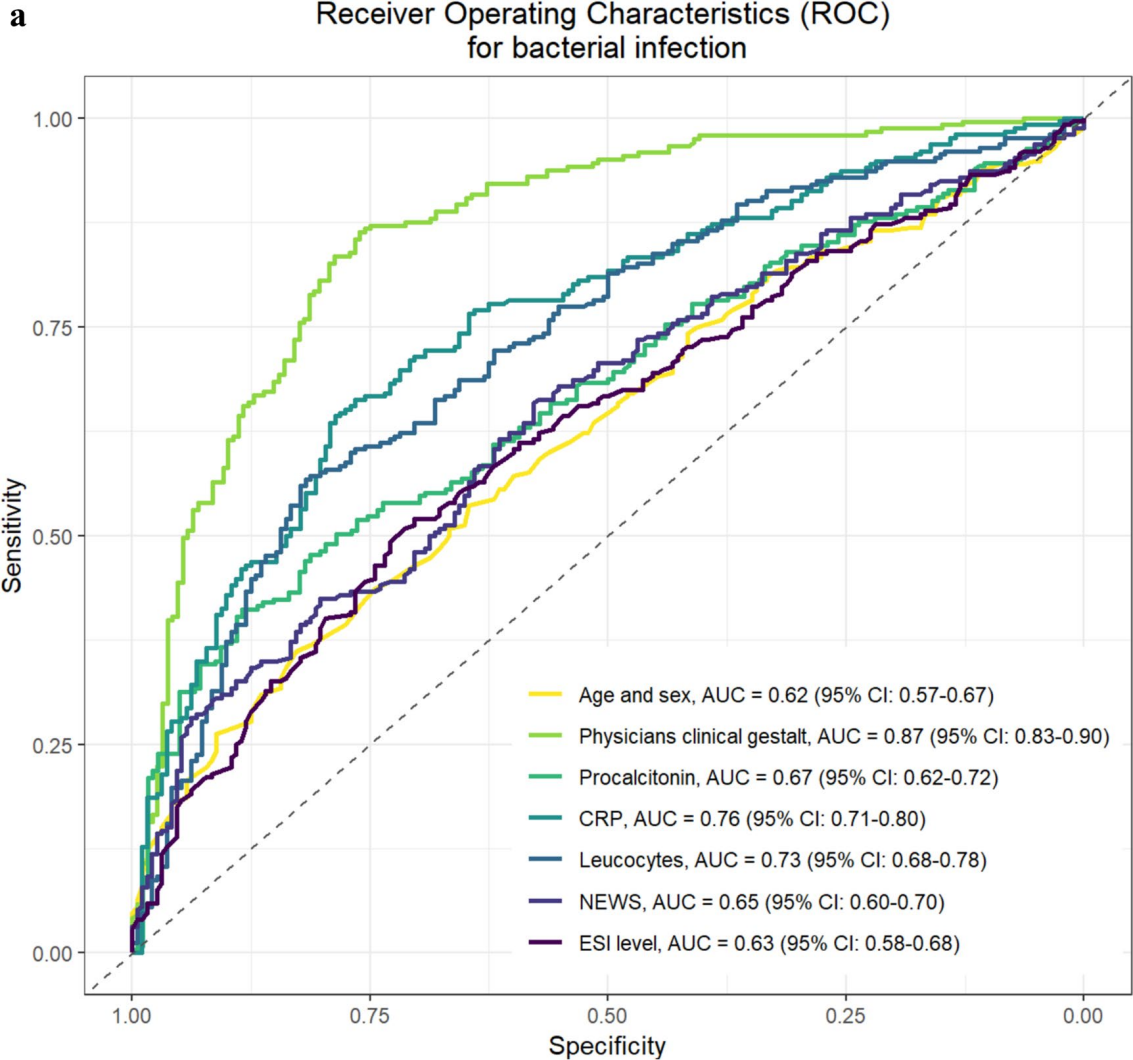

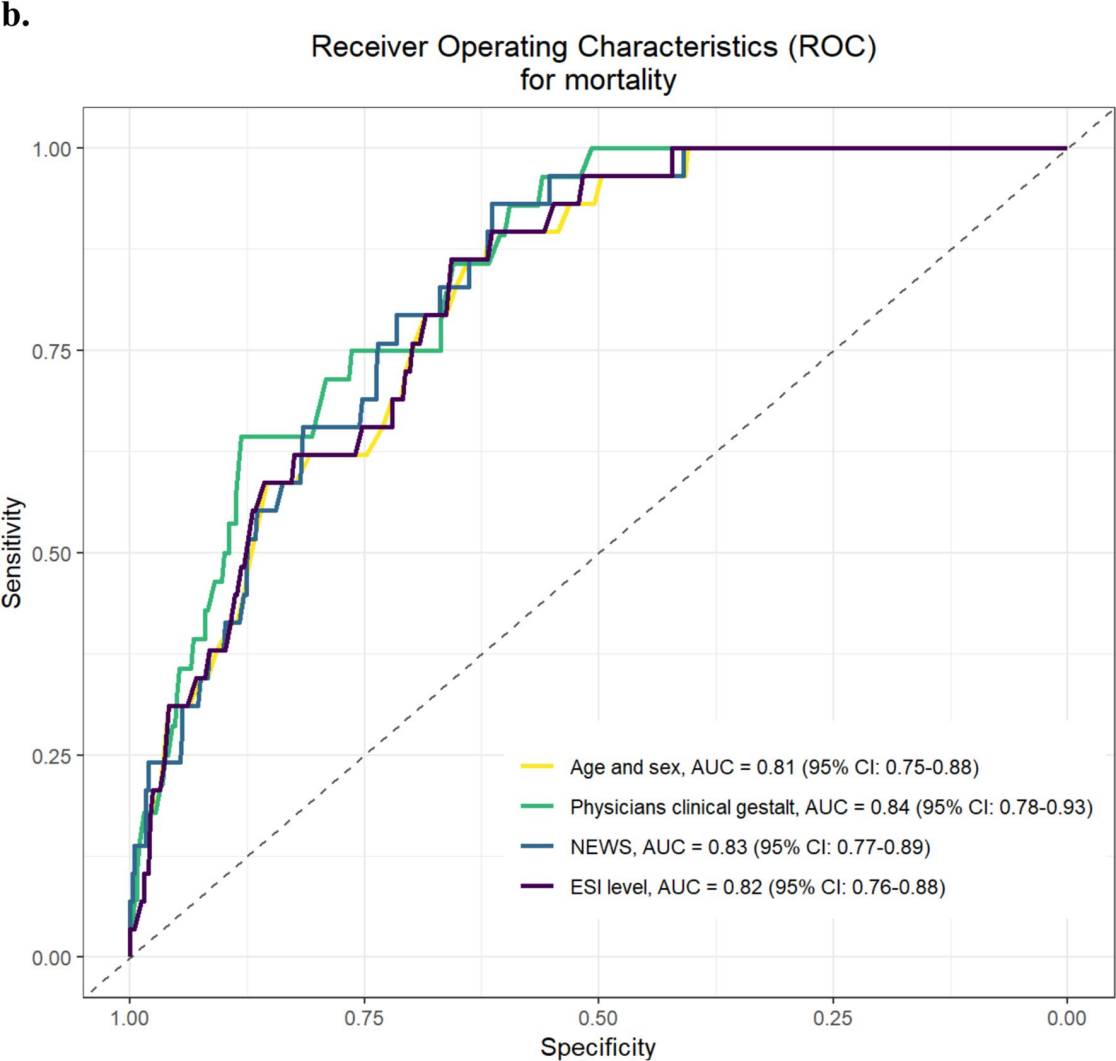
Receiver operating characteristics (ROC) curves with AUCs. **a** ROC curves with areas under the curve (AUCs) for bacterial infection. ROC for bacterial infection adjusted for age and sex. AUC with corresponding confidence interval (CI) are printed. Abbreviations: AUC, area under the curve; CI, confidence interval; CRP, C-reactive protein; ESI, Emergency Severity Index; NEWS, National Early Warning Score; ROC, receiver operating characteristics. **b** ROC curves with AUCs for 30-day mortality. ROC for 30-day mortality adjusted for sex and age. Areas under the curves with corresponding confidence interval (CI) are printed. Abbreviations: AUC, area under the curve; CI, confidence interval; ESI, Emergency Severity Index; NEWS, National Early Warning Score; ROC, receiver operating characteristics.

The AUCs of the ROC analysis for the outcome “bacterial infection,” after adjustment for age and sex, were the following: 0.87 [95%CI 0.83 to 0.90] for clinical gestalt, 0.67 [95%CI 0.62 to 0.72] for PCT, 0.76 [95%CI 0.71 to 0.80] for CRP, 0.73 [95%CI 0.68 to 0.78] for leucocytes, 0.65 [95%CI 0.60 to 0.70] for NEWS, and 0.63 [95%CI 0.58 to 0.68] for ESI (Fig. 2a). The AUC of clinical gestalt was higher than the tested inflammatory markers and scores (all *p*-values < 0.001). The results of the sensitivity analysis only including high adjudication certainty cases were comparable (Appendix Figure 3). After adjustment for age and sex, the OR from the logistic regression model for bacterial infection was 1.69 [95%CI 1.55 to 1.86] per one-unit increase on the VAS of the clinical gestalt regarding bacterial infection likelihood (Table 3).

**Table 3:** Odds Ratios (OR) from Logistic Regression Model

	Bacterial infection		30-day mortality	
	*OR [95% CI]*	*P-value*	*OR [95% CI]*	*P-value*
	**Without adjustment**			
Female sex	1.26 [0.76–2.13]	*0.370*	0.33 [0.11–0.84]	*0.028*
Age, per year	1.01 [1.00–1.03]	*0.042*	1.08 [1.04–1.12]	*<0.001*
One-unit increase on the VAS of the physicians’ clinical gestalt regarding bacterial infection likelihood	1.69 [1.55, 1.86]	*<0.001*	-	-
Physician’s answer to the mortality probability question is yes	-	-	6.71 [3.05–15.14]	*<0.001*
	**Adjusted for age and sex**			
One-unit increase on the VAS of the physicians’ clinical gestalt regarding bacterial infection likelihood	1.69 [1.55–1.86]	*<0.001*	-	*-*
Physician’s answer to the mortality probability question is yes	-	*-*	3.40 [1.47–8.01]	*0.004*

OR for diagnosis of bacterial infection and 30-day mortality, calculated with logistic regression models

*Abbreviations*: *CI*, confidence interval; *OR*, odds ratio; *VAS*, visual analog scale

### Antibiotic Treatment

In the group of patients with a bacterial infection, 226 (89.7%) received antibiotic treatment within 24 h; 31 (27.4%) patients with viral infections, 2 (66.7%) patients with other infections, and 16 (21.1%) patients with no infection received antibiotics (Table 2). Physicians’ clinical gestalt regarding bacterial infection likelihood was 8.0 [IQR 6.4, 9.0] of 10 in patients treated with antibiotics and 2.2 [IQR 1.0, 4.6] of 10 in the group not receiving antibiotics (*p*-value = <0.001) (Appendix Table 5).

### Prediction of 30-Day Mortality

In the 30-day non-survivors, 16 (55.2%) were judged to have a high likelihood of dying and 12 (41.1%) were judged to have a low likelihood of dying (*p*-value < 0.001) (Appendix Table 6). The clinical gestalt’s sensitivity regarding mortality was 57.1% [95%CI 37.2 to 75.5] with a specificity of 83.4% [95%CI 79.7 to 86.9]. The positive LR was 3.45 [95%CI 2.33 to 5.09] and the negative LR 0.51 [95%CI 0.33 to 0.79] (Appendix Table 7).

The AUCs of the ROC analysis regarding high likelihood of death, after adjustment for age and sex, were 0.84 [95%CI 0.78 to 0.93] for clinical gestalt, 0.83 [95%CI 0.77 to 0.89] for NEWS, and 0.82 [95%CI 0.76 to 0.88] for ESI (Fig. 2b). After adjustment for age and sex, the OR derived from the logistic regression model for 30-day mortality was 3.40 [95%CI 1.47 to 8.01] (Table 3).

## DISCUSSION

The main results of this prospective observational study of patients presenting to the ED with signs and symptoms of infection were the following: First, ED physicians’ clinical gestalt was shown to be more accurate than inflammatory markers such as CRP, PCT, and leucocyte count, as well as scores as the NEWS and ESI, in predicting bacterial infections. Second, patients with a high likelihood of bacterial infection according to clinical gestalt had higher antibiotic prescription rates than those with viral or no infections. Third, simple heuristic prognostication of mortality (likely vs. unlikely) by ED physicians had some, but limited, prognostic value.

Previous studies have shown that physicians’ clinical impression is associated with the severity of sepsis, and that it stratifies between patients requiring admission to intensive care or a general ward.^26,27^ The initial suspicion of sepsis, however, was based on clinical tools such as quick Sequential Organ Failure Assessment (qSOFA) score, which can be rapidly performed and easily guide the physician’s clinical impression. No such tool exists for detecting bacterial infections from infections by other pathogens. The superiority of a fast and heuristic pattern recognition (“gestalt”) by emergency physicians against well-established inflammatory markers and scores merits discussion.

While inflammatory markers have shown some value in the screening of infection, their ability to differentiate between viral and bacterial infections is discussed controversially.^36–38^ The identification of patients requiring early antibiotic treatment in the ED is a challenge. According to our data, almost 90% of patients with a final diagnosis of bacterial infection received early antibiotic therapy, while about 20% of patients with early antibiotic therapy had no infection or no bacterial infection. Antibiotic treatment without bacterial infection is lower than in comparable studies.^39^ Reasons for this finding may be the multiple studies and programs performed at our hospital over many years,^39,40^ as well as the rising general awareness of inappropriate antibiotic use. Among other issues, antibiotics are associated with adverse events and antimicrobial resistance.^41^ On the other hand, the early initiation of antibiotic treatment improves the prognosis of ED patients at risk of sepsis.^28,29,42,43^ By using gestalt in patients with suspected infection at presentation, appropriate and early antibiotic therapy could be improved, particularly in EDs with triage liaison physicians. ^32^ However, this is a pilot study testing the hypothesis that clinicians can accurately gauge the likelihood of bacterial infection, which is the main prerequisite for bacterial sepsis. These results are promising, considering that there is also a good positive correlation between clinical gestalt regarding bacterial infection and early antibiotic therapy. To determine whether the question “how likely is bacterial infection” during triage or first clinical assessment in the ED could improve outcomes in patients with possible infection, an interventional study should be conducted using “usual care” as comparator. The present data can be used as background for such a venture, but not yet as “proof of principle.” Other tools for sepsis detection have already been introduced into clinical routine, but the success of implementation is rather modest. ^16^ While tools such as sepsis scores have their benefits, a major problem is their dependence on data not readily available at presentation. ^44^ On the other hand, clinical gestalt, if implemented in a structured manner, may also be used as a valuable tool for prognostication of bacterial infection and sepsis. This new knowledge could ultimately lead to the development of more accurate tools. Such tools may consist of gestalt assessment combined with parameters readily available at triage, such as vital signs, symptoms, or assessments of speech and gait.^18,45–48^

Regarding mortality prediction, our findings are in line with other studies showing that clinical gestalt has some value in identifying patients at risk for mortality.^49,50^ In addition, our results are consistent with the findings of previous studies concerning the high negative predictive value (NPV) of clinical gestalt.^51^ In contrast to the cited study with slightly higher positive predictive value (PPV), we assessed the physicians’ gestalt at ED presentation and not at admission to the hospital to identify patients at risk at the earliest possible time point to trigger additional assessment, treatment options, and adapt resource allocation.^52,53^

### Strength and Limitations

A strength of this study is its prospective and observational design, conducted at a tertiary care center. The rigorous adjudication of infection status by an expert panel and the integration of clinical gestalt assessment alongside inflammatory markers offer a comprehensive evaluation of their predictive utility in real-world ED settings.

However, we are also aware of some limitations. First, this single-center study was conducted in a mostly Caucasian population. The high percentage of high-acuity patients, as shown by ESI levels, may not be representative of all EDs, as this can vary depending on the healthcare system and referral patterns. However, the low percentage of low-acuity patients and high hospitalization rates are typical for urban central European EDs. The questions were asked after the first contact with the patient. Data available to the physician were not documented. Because ED physicians did not give informed consent, we could not assess possible confounders such as physicians’ age, gender, or experience, but it has previously been shown that experience directly correlates with accuracy of physicians’ clinical gestalt.^54–56^ Additionally, the present study did not use the often used “surprise question”— “Would you be surprised, if your patient died in the next month?”— but focused on high versus low probability of death.^57,58^ Despite using a standardized modified Delphi process, the adjudication of final diagnoses has its inherent limitations and involves subjectivity, particularly in cases without positive microbiological tests. Thus, interrater reliability was suboptimal. Missing data in our study were due to early discharge or transfer (16.2%), missing blood samples (8.5%), and loss to follow-up (0.7%). These missing data were largely unrelated to clinical outcomes or patient characteristics, reducing the likelihood of systematic bias. Another limitation is the lack of information on race and income. However, our cohort is predominantly of Caucasian origin and health-insured.

## CONCLUSION

In this prospective study in ED patients presenting with signs and symptoms of infection, clinical gestalt in form of a likelihood assessment of bacterial infection was shown to be more accurate than CRP, PCT, leucocyte counts, NEWS, and ESI in differentiating between bacterial infection and infection of other causes, or no infection. In patients with a high likelihood of bacterial infection, according to the physician’s gestalt, antibiotic prescription rates were higher than in patients without infections and in patients with viral infections. Lastly, simple heuristic prognostication of mortality (likely vs. unlikely) by physicians has some, but limited, prognostic value.

## Supplementary Information

Below is the link to the electronic supplementary material.

Supplementary file1 (DOCX 24 KB)

## Data Availability

The data supporting the findings of this study are available upon reasonable request. Access to the data is granted to individuals who can provide a valid authorization from an ethics committee. Requests for data access can be made via email to roland.bingisser@usb.ch.

## Abbreviations

AUC: Area under the curve
CRP: C-reactive protein
ED: Emergency department
ELISA: Enzyme-linked immunosorbent assay
ESI: Emergency Severity Index
IQR: Interquartile range
PCT: Procalcitonin
ROC: Receiver operating characteristics
SIRS: Systemic inflammatory response syndrome
SOFA: Sequential Organ Failure Assessment
NEWS: National Early Warning Score
VAS: Visual analog scale
NPV: Negative predictive value
PPV: Positive predictive value
MEWS: Modified Early Warning Score
