# Supplementary material for: Clinical Gestalt to Predict Bacterial Infection and Mortality in Emergency Department Patients: A Prospective Observational Study
Source: J Gen Intern Med. 2025 Feb 26;41(1):73–82. doi: 10.1007/s11606-025-09440-7 (PMC12855699; doi:10.1007/s11606-025-09440-7)
Supplement: Supplementary file 1 — Supplementary file1 (DOCX 24 KB) [file 11606_2025_9440_MOESM1_ESM.docx]

**Appendix**

**Table 4** Baseline Characteristics, Inflammatory Marker Levels, and Outcomes According to Adjudication Method

|  | **Microbiological test** | **Expert consensus** | **Referee decision** |
| --- | --- | --- | --- |
| ***n* (%)** | 201 (45.3) | 174 (39.2) | 69 (15.5) |
| **Median age, median [IQR]** | 70 [55, 79] | 68 [49, 80] | 64 [50, 79] |
| **Female sex, *n* (%)** | 94 (47) | 72 (41) | 29 (42) |
| **ESI triage level** |  |  |  |
| ESI 1, *n* (%) | 2 (1.0) | 3 (1.7) | 1 (1.4) |
| ESI 2, *n* (%) | 111 (55.2) | 99 (56.9) | 38 (55.1) |
| ESI 3, *n* (%) | 85 (42.3) | 70 (40.2) | 30 (43.5) |
| ESI 4, *n* (%) | 3 (1.5) | 2 (1.1) | 0 (0.0) |
| ESI 5, *n* (%) | 0 (0.0) | 0 (0.0) | 0 (0.0) |
| **NEWS, median [IQR]** | 5 [3, 8] | 5 [3, 7] | 5 [3, 7] |
| **Inflammatory markers** |  |  |  |
| Serum CRP (mg/l), median [IQR] | 70.8 [26.0, 176.1] | 34.7 [12.1, 112.0] | 70.9 [31.5, 150.3] |
| Serum procalcitonin (ng/ml), median [IQR] | 0.3 [0.1, 1.5] | 0.1 [0.1, 0.3] | 0.1 [0.1, 0.4] |
| Leucocyte count (×10^9^/l), median [IQR] | 10.5 [7.3, 14.4] | 10.7 [7.9, 13.6] | 11.0 [7.6, 14.8] |
| **Infection status** |  |  |  |
| No infection, *n* (%) | 0 (0) | 60 (34.5) | 16 (23.2) |
| Bacterial infection, *n* (%) | 122 (60.7) | 75 (43.1) | 30 (43.5) |
| Bacterial and viral co-infection, *n* (%) | 19 (9.5) | 1 (0.6) | 5 (7.2) |
| Viral infection, *n* (%) | 58 (28.9) | 38 (21.8) | 17 (24.6) |
| Other infection, *n* (%) | 2 (1.0) | 0 (0.0) | 1 (1.5) |
| **Physicians’ clinical gestalt regarding bacterial infection likelihood (VAS, 0–10), median [IQR]** | 7.3 [2.7, 8.9] | 5.5 [1.9, 7.9] | 6.8 [3.6, 8.2] |
| **Outcomes** |  |  |  |
| 30-day mortality, *n* (%) | 13 (6.5) | 12 (6.9) | 4 (5.8) |

Baseline characteristics, inflammatory markers levels and outcomes according to adjudication method. Data are reported as median [IQR] or *n* (%)

*Abbreviations*: *CRP*, C-reactive protein; *ESI*, Emergency Severity Index; *ICU*, intensive care unit; *IQR*, interquartile range; *NEWS*, National Early Warning Score; *VAS*, visual analog scale

**Table 5** Comparison of Patients Separated by Patients Treated with Antibiotic or Not

|  | **Antibiotic treatment** | **No antibiotic treatment** | ***P*-value** |
| --- | --- | --- | --- |
| ***n* (%)** | 275 (61.9) | 169 (38.1) |  |
| **Infection status** |  |  | <0.001 |
| No acute infection, *n* (%) | 16 (5.8) | 60 (35.5) |  |
| Bacterial infection, *n* (%) | 226 (82.2) | 26 (15.4) |  |
| Viral infection, *n* (%) | 31 (11.3) | 82 (48.5) |  |
| Other infection, *n* (%) | 2 (0.7) | 1 (0.6) |  |
| **Physicians’ clinical gestalt regarding bacterial infection likelihood, VAS (0–10)** | 8.0 [6.4, 9.0] | 2.2 [1.0, 4.6] | <0.001 |

Data are reported as median [IQR] or *n* (%)

*Abbreviations*: *IQR*, interquartile range; *VAS*, visual analog scale

**Table 6** Comparison of Patients by 30-Day Mortality

|  | **30-day survivors** | **30-day non-survivors** | ***P*-value** |
| --- | --- | --- | --- |
| ***n* (%)** | 412 (93.4) | 29 (6.6) |  |
| **Age, median [IQR]** | 67 [51, 79] | 83 [79, 87] | <0.001 |
| **Female sex, *n* (%)** | 188 (45.6) | 6 (20.7) | 0.015 |
| **NEWS, median [IQR]** | 5 [3, 7] | 7 [5, 8] | <0.001 |
| **Infection status** |  |  | 0.056 |
| No acute infection, *n* (%) | 72 (17.5) | 4 (13.8) |  |
| Bacterial infection, *n* (%) | 226 (54.9) | 23 (79.3) |  |
| Viral infection, *n* (%) | 111 (26.9) | 2 (6.9) |  |
| Other infection, *n* (%) | 3 (0.7) | 0 |  |
| **Physicians’ clinical gestalt regarding probability of patient’s death in the next month** |  |  | <0.001 |
| Yes, *n* (%) | 66 (16.0) | 16 (55.2) |  |
| No, *n* (%) | 332 (80.6) | 12 (41.4) |  |
| Missing value, *n* (%) | 14 (3.4) | 1 (3.4) |  |

Data are reported as median [IQR] or *n* (%)

*Abbreviations*: *IQR*, interquartile range; *NEWS*, National Early Warning Score

**Table 7** Discriminative Performance of Physicians’ Clinical Gestalt on 30-Day Mortality

|  | **Value** | ***95% CI*** |
| --- | --- | --- |
| **Sensitivity** | 57.1% | *[37.2 ,75.5]* |
| **Specificity** | 83.4% | *[79.7, 86.9]* |
| **Positive likelihood ratio** | 3.45 | *[2.33, 5.09]* |
| **Negative likelihood ratio** | 0.51 | *[0.33, 0.79]* |
| **Positive predictive value** | 19.5% | *[14.1, 26.3]* |
| **Negative predictive value** | 96.5% | *[94.7, 97.7]* |

**Fig. 3** Sensitivity analysis — receiver operating characteristics (ROC) curves with AUCs for bacterial infection of the patients with very high adjudication certainty. ROC for bacterial infection. Areas under the curves (AUCs) with corresponding confidence interval (CI) are printed. Abbreviations: AUC, area under the curve; CI, confidence interval; CRP, C-reactive protein; ESI, Emergency Severity Index; NEWS, New Early Warning Score; ROC, receiver operating characteristics
